# Novel flow cytometry technique for detection of *Plasmodium falciparum* specific B-cells in humans: increased levels of specific B-cells in ongoing infection

**DOI:** 10.1186/s12936-015-0911-0

**Published:** 2015-09-26

**Authors:** Allan Lugaajju, Sreenivasulu B. Reddy, Caroline Rönnberg, Mats Wahlgren, Fred Kironde, Kristina E. M. Persson

**Affiliations:** School of Biomedical Sciences, College of Health Sciences, Makerere University, Kampala, Uganda; Habib Medical School, Islamic University in Uganda, Kampala, Uganda; Microbiology, Tumor, and Cell Biology, Karolinska Institutet, Solna, Sweden; Department of Laboratory Medicine, Lund University, Lund, Sweden

**Keywords:** *Plasmodium falciparum*, Quantum dots, Ghost infected red blood cells, Malaria, B-cells

## Abstract

**Background:**

Malaria caused by *Plasmodium falciparum* is still a major health threat in endemic areas especially for children below 5 years of age. While it is recognized that antibody immunity plays an important role in controlling the disease, knowledge of the mechanisms of sustenance and natural boosting of immunity is very limited. Before, it has not been possible to investigate malaria specific B-cells directly in flow cytometry, making it difficult to know how much of a B cell response is due to malaria, or how much is due to other immunological stimulators.

**Methods:**

This study developed a technique using quantum dots and schizont extract made from ghosts of infected erythrocytes, to be able to investigate *P. falciparum* specific B-cells, something that has never been done before.

**Results:**

Major differences in *P. falciparum* specific B-cells were found between samples from immune (22.3 %) and non-immune (1.7 %) individuals. Samples from parasite positive individuals had the highest proportions of specific B-cells (27.9 %).

**Conclusion:**

The study showed increased levels of *P. falciparum*-specific B-cells in immune individuals, with the highest levels in active malaria infections, using a new technique that opens up new possibilities to study how these cells are sustained in vivo after natural infections. It will also be useful in vaccine studies.

## Background

Clinical immunity to *Plasmodium falciparum* malaria is acquired and maintained by repeated exposure to the parasite [1]. Classical studies have demonstrated that passively transferred IgG from semi-immune adults with repeated prior exposure to *P. falciparum* infection can clear or reduce parasitaemia in individuals acutely infected with *P. falciparum* [2]. However, the mechanisms by which antibody-secreting cells are induced and maintained for long-term disease protection are poorly understood. In order to appreciate how *P. falciparum* specific B-cells are induced and maintained in vivo, these cells need to be separated from other B-cells. The maintenance of serum antibody levels after exposure to antigen either by infection or immunization has been referred to as serological memory [3]. There is long-standing evidence that naturally acquired immunity to the erythrocytic stages of malaria is strongly dependent on antibodies [4–8]. Memory B-cells play an important role in memory for different pathogens, by boosting the immune response in times of secondary exposure. Naturally the kinetics of antibodies is a balance between production and decay. Studies have shown that production of antibodies against merozoite antigens is not sustained following an acute episode of malaria [4, 9]. Antibody production can be sustained through restimulation of memory B-cells by persistent antigens [10] or by non-proliferating long lived plasma cells [11]. The mechanisms mediating the development of short-lived antibody secreting cells and long lived plasma cells are not well understood. However, it appears that short-term serological memory may be dependent on antigen stimulation whereas long-term serological memory is antigen independent and depends on homeostatic activation. Studies have shown that during acute malaria infection, there are acute alterations in memory B-cell numbers [12]. However, these studies used the entire B-cell population without differentiating whether they were *P. falciparum* specific or not.

Controversy still prevails as to why memory in human malaria infections is short-lived [13]. Antibody levels to some malarial antigens, although not all, have been found to rapidly decline after the end of the transmission season and it has been shown that immunity is short-lived in the absence of reinfection [14–19]. This implies that B-cell memory to malaria may be defective or suboptimal. Weiss et al. [20] provided evidence that an atypical memory B-cell population is significantly expanded in *P. falciparum*-exposed Malian adults and children as young as 2 years of age. While atypical and classical memory B-cells appear closely related developmentally, atypical memory B-cells exhibit markedly reduced signaling and effector functions, which may contribute to the inefficient acquisition of humoral immunity to malaria [21].

Some studies in animal models have shown that memory B-cells do develop and are maintained normally after malaria infections [22, 23], whereas others have found that malaria infection interferes with the development of memory B-cells and long-lived plasma cells [24, 25]. In humans, several studies have demonstrated stable antibody responses to malaria antigens [26–28]. However, short-lived antibody responses have also been observed [29, 30], especially in young children [27]. Dorfman et al. [30] were frequently unable to detect circulating malaria-specific B-cells in seropositive children, but it is unclear whether this reflects an absence of such cells or a lack of sensitivity in the assays used to detect them. Nahrendorf et al. [31] showed gradual acquisition of memory B-cells and antibodies recognizing pre-erythrocytic and cross-stage antigens after *P. falciparum* sporozoite immunization. However, the magnitude of these humoral responses did not correlate with protection but directly reflected parasite exposure in chemoprophylaxis and sporozoites immunization and challenge. Asito et al. [12] observed an increase in both the total memory B-cell population and the transitional B-cell population, following an episode of acute malaria in African children. However, this study lacked any analysis of the specificity of B-cell responses as well as long-term follow up to ascertain the duration of the response. One study showed that even if antigen-specific antibodies were not detected in plasma, antigen-specific B-cells could still be found circulating in the blood, suggesting that these could be maintained independently of long-lived plasma cells [32]. Most of these studies used Elispot assays for the detection of antigen specific memory B-cells. It has been suggested before that flow cytometry is a good method for estimation of antigen-specific cells [33] in situations with complex antigens, compared to ELISA-based assays, and malaria is certainly a case where there are several setups of antigens, containing a range of merozoite, sporozoite and infected erythrocyte antigens where the concentration of each specific B-cell is often very low. Even though ELISA-based assays can be made more sensitive through different measures, it is still estimated that they might detect only 70 % of the response found when using flow cytometry [33]. An advantage of using direct flow cytometry, as in the method described here, is that no stimulation of the cells is needed, increasing the chances of including all cells in the reading. When this method was used in a set of immune and non-immune donors, very clear differences were found between the two groups, with the highest levels in on-going malaria infection.

## Methods

### Sample collection and processing

Samples from malaria endemic (n = 57) and non-endemic areas (n = 25) were collected from Kasangati Health Centre, Uganda, and Karolinska University Hospital (blood donors), respectively. All samples from Kasangati, except seven of the RDT positive samples, were collected during the high transmission season. Additionally, five samples with an inflammatory condition other than malaria were collected during the low transmission season. 10 Samples each from Ugandan and Swedish donors were also collected for protocol standardization. Written informed consent was obtained from all study participants in Uganda and the study protocol was approved by the appropriate Ethical review committee (Uganda: 2011-114, Sweden: 2014-478-32). Blood (5–10 mL) was drawn in lithium heparinized tubes (BD, Plymouth, UK) and transported to the laboratory for processing within 4 h after collection. Peripheral blood mononuclear cells (PBMC) were separated by Ficoll-hypaque (GE HealthCare Bio-Sciences AB, Sweden) density gradient centrifugation. The sample blood was diluted with equal volumes of Dulbecco’s phosphate buffered saline (DPBS, Life technologies, Stockholm, Sweden), carefully layered over the Ficoll, and centrifuged for 30 min at 400 g (room temperature) with no brakes. After centrifugation, whole blood was separated into four layers, with plasma at the top followed by white blood cells containing PBMCs, a Ficoll medium layer, and a bottom layer containing erythrocytes and granulocytes. After the removal of the plasma, the PBMCs were carefully collected, and washed twice with DPBS at 400 g for 15 min, then 300 g for 15 min to remove Ficoll traces and platelets. The cells were then resuspended in 1 mL RPMI media (Sigma, St Louis, MO, USA). The Cell count was done with the Neuberger counting chamber (5 μL of the cell suspension was added to 45 μL of 0.4 % trypan blue, Sigma, St Louis, MO, USA). The PBMCs were cryopreserved in liquid nitrogen at a concentration of 10^7^ cells/mL in heat-inactivated 90 % fetal bovine serum (Sigma, St Louis, MO, USA) and 10 % DMSO (Sigma, St Louis, MO, USA) as previously described [34].

### *Plasmodium falciparum* infected red blood cells (iRBCs)

*Plasmodium falciparum*-infected red blood cells of the FCR3S 1.2 line were maintained in vitro at pH 7.4 in sealable flasks using human group O+ erythrocytes, at 3 % haematocrit, in RPMI-HEPES medium supplemented with 50 μg/mL hypoxanthine, 25 mM NaHCO_3_, 20 μg/mL gentamicin, 5 % (vol/vol) heat-inactivated pooled human sera from donors resident in Sweden, and 0.25 % Albumax II (Gibco, Invitrogen, Mount Waverly, Australia) maintained in an atmosphere of 1 % O_2_, 4 % CO_2_, and 95 % N_2_ at 37 °C, as previously described [35]. Cultures were synchronized two or three times per week by resuspending culture pellets in 5 % d-sorbitol (Sigma, St Louis, MO, USA) in water to lyse trophozoite- and schizont-infected erythrocytes.

### Ghost *Plasmodium falciparum* infected red blood cells (GiRBCs)

The pellet of 1 × 10^8^ to 5 × 10^8^ enriched trophozoite stage parasites (~80 % parasitaemia) was treated with Streptolysin O (SLO) from Sigma to obtain GiRBCs following the protocol in [36]. SLO treatment of infected erythrocytes results in the release of the erythrocyte cytosol. Parasites contained within an intact parasitophorous vacuole were sedimented by centrifugation at 10,000×*g* for 15 s. The pellet (GiRBC) was then homogenized using a sonicator (Q500, Fisher Scientific), and the protein concentration was measured using a Nanodrop (ND2000, Thermoscientific).

### Quantum dot (Qdot) conjugation of iRBCs and GiRBCs

#### Amino Qdot conjugation

35 µL of 2 nmol Qdot 565 amino Qdot (Invitrogen) was transferred into a clean dry glass vial. 3.5 µL of 1 mM *Bis*[sulfosuccinimidyl] suberate (BS3) was added and incubated for 30 min on a rotator. The Qdots were purified from excess cross-linker by buffer exchange on a clean centrifugal ultra filtration unit (Millipore Corporation), which was pre-equilibrated with 3000 µL of PBS (pH 7.4). The collected eluent was added to a clean dry vial containing 225 µg of iRBC/GiRBC and mixed gently, then allowed to react for 2 h at room temperature on a rotator. The reaction was quenched with 1 M glycine to a final concentration of 50 mM for 15 min. The conjugate solution was filtered through a 0.2 µm poly ether sulfone (PES) syringe filter over a clean centrifugal ultra filtration unit (Millipore: 100 kDa cutoff). The filtrate was washed thrice with 3 mL of 50 mM borate buffer (pH 8.3) at 3000 g for 5 min per wash. The amino Qdot-iRBC/GiRBC conjugate solution was diluted 10 times with 10 mM Borate buffer (pH 7.4) and stored at 4 °C until further processing.

#### Carboxyl Qdot conjugation

35 µL of 2 nmol Qdot 565 carboxyl Qdots (Invitrogen) was transferred into a clean dry glass vial. 199 µL of 10 mM borate buffer (pH 7.4) was then added. 225 µg of iRBCs/GiRBC was added followed by addition of 7 µL freshly prepared 10 mg/mL *N*-ethyl-*N*′-dimethylaminopropyl-carbodiimide (EDC). The reaction mixture was then incubated for 2 h at room temperature with gentle stirring. The conjugate solution was filtered, washed, and diluted as described above for Amino Qdot conjugation.

### Immunophenotyping of *P. falciparum* specific B-cells

Cryopreserved PBMCs (approximately 1 × 10^6^ cells) were thawed on ice and washed in cold flow buffer (PBS/0.5 % BSA/2 mM EDTA). To each sample, 100 µL flow buffer was added. To remove non-specific binding and background fluorescence, 1 µg Fc block (CD16/CD32mAb, Biolegend) was added to each PBMC sample, and incubated on ice for 5 min. 25 µL of GiRBC-Qdot conjugate was added, and incubated on ice for 30 min. After incubation, the cells were washed and stained for CD19 B-cell phenotyping. 3.5 µL CD 19 PE CF594 fluorochrome-conjugated mouse antihuman mAb (BD Horizon) was used to stain 10^6^ cells/100 µL flow buffer. After staining for 30 min, cells were washed with flow buffer and resuspended in 300 µL flow buffer. Analysis was done on a LSRII flow cytometer (Becton–Dickinson Immunocytometry Systems, San Jose, USA). Data was processed using FLOWJO software (Tree Star Inc., San Carlos, CA, USA).

### Malaria diagnostics

All samples from Uganda were subjected to a Combo Rapid Diagnostic Test (pLDH/HRP2, Premier Medical Corporation Limited, India) as described [37]. The parasitaemia for the malaria positive samples was calculated using microscopy according to the WHO guide lines [38].

## Results

### Comparison between amino Qdots and carboxyl Qdots

**Fig. 1:**
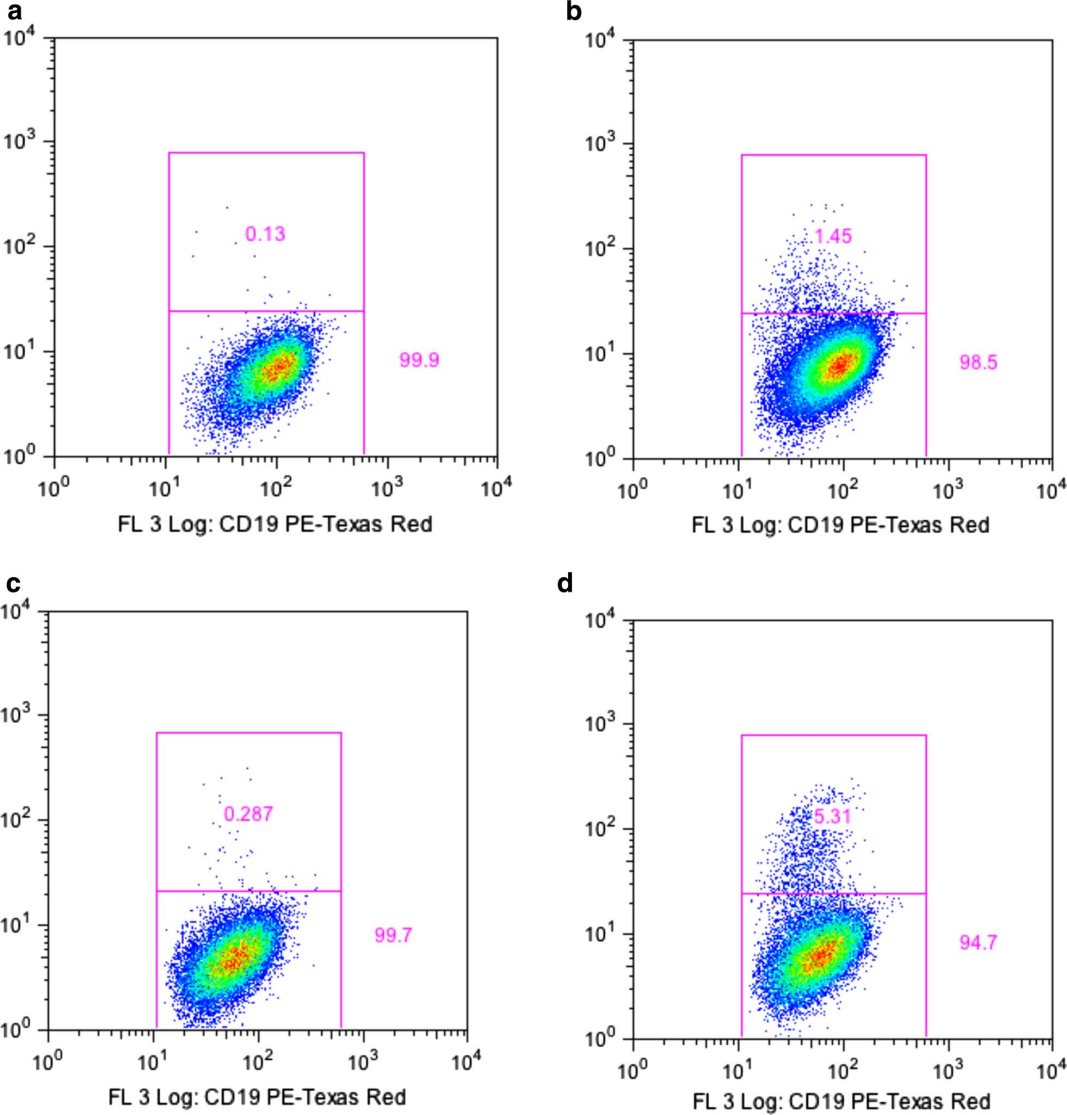
Representative flow cytometry experiment from comparison of amino Qdot-iRBCs and carboxyl Qdots-iRBCs conjugate. Immunophenotypying of both PBMCs samples from malaria endemic (immune) and malaria non-endemic (non immune) areas was done using amino Qdot-iRBC/carboxyl Qdot-iRBC, and CD 19 immunophenotyping based on carboxyl Qdot-iRBC gave a better separation of falciparum+ B-cells between immune and non-immune samples. **a**, **c** Non-immune and immune samples immunophenotyped by amino Qdot-iRBCs; **b**, **d** non-immune and immune samples immunophenotyped by carboxyl Qdot-iRBCs

To establish the optimal volume of amino/carboxyl Qdot conjugate that reacts with the PBMC samples, the volumes of both amino and carboxyl iRBC conjugates were varied (from 10, 15, 20, 25, 30, 35, and 40 µL) on the same samples from malaria endemic and non-endemic areas. 25 µL of the conjugate (containing 1 µL Qdot: 6.4 μg iRBC/GiRBC) was found to be optimal. Furthermore, immune and non immune immunophenotyping based on carboxyl Qdot-iRBC conjugate gave a higher *P. falciparum* positive frequency (even though the background was also higher), as compared to the amino Qdot-iRBCs conjugate, implying that carboxyl Qdots are better separators for *P. falciparum* specific B-cells compared to amino Qdots (Fig. 1). The mean % of falciparum+ B-cells as determined by amino and carboxyl Qdots was 0.27 and 5.38, respectively, with a p value of <0.0001. The difference between % of falciparum+ B-cells as determined by amino and carboxyl Qdots was statistically significant.

### Comparison of carboxyl Qdot-iRBC and carboxyl Qdot-GiRBC

Despite carboxyl Qdot-iRBC being better separators of *P. falciparum* specific B-cells, they have one short-fall of unspecific binding. To reduce on the unspecific binding of carboxyl Qdot-iRBC conjugate to haemoglobin, the GiRBC (which eliminates most of the haemoglobin) was prepared and conjugated with carboxyl Qdots as described above.

**Fig. 2:**
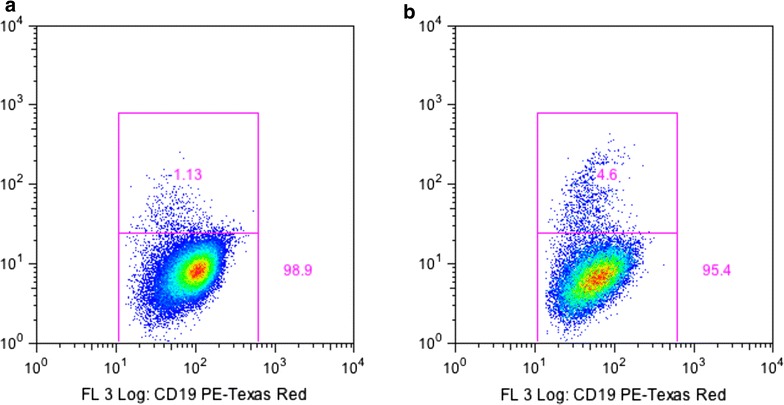
Example of comparison in flow cytometry of carboxyl Qdot-iRBC and carboxyl Qdot-GiRBC for the same Ugandan blood donor, showing a higher % of falciparum+ CD19-positive B-cells for GiRBC. **a** Immunophenotyping with carboxyl Qdot-iRBC, **b** immunophenptyping with carboxyl Qdot-GiRBC

Immunophenotyping experiments to compare carboxyl Qdot-iRBC with carboxyl Qdot-GiRBC on four different Ugandan blood donors showed that carboxyl Qdot-GiRBC gave a higher *P. falciparum* positive percentage as depicted in Fig. 2, implying that carboxyl Qdot-GiRBC is better than carboxyl Qdot-iRBC.

The mean % of falciparum+ B-cells as determined by carboxyl Qdot-RBC and carboxyl Qdot-GiRBC was 1.2 and 4.6, respectively. The difference between % of falciparum+ B-cells as determined by carboxyl Qdot-RBC and carboxyl Qdot-GiRBC was statistically significant with p value of <0.0001.

### Comparison of fresh and frozen PBMC

To establish the effect of cryopreservation on PBMC using this method, two blood samples from non-immune donors were used. The PBMC were isolated from each sample using ficoll hypaque as described above. Part of the PBMC (fresh) from each sample was immediately immunophenotyped (carboxyl Qdot GiRBC and CD 19 PE CF594 fluorochrome-conjugated mouse antihuman mAb) as described above. The remaining portion (frozen) was cryopreserved and immunophenotyped the same way as the fresh PBMC after 1 week. One sample showed 1.7 % falciparum+ B-cells when fresh and 1.6 % after having been frozen, and the other sample showed 2.2 % both as fresh and after freezing. Hence the freezing of PBMC has no effect on this technique.

### Separation of falciparum+ B-cells from falciparum− B-cells using carboxyl Qdot-GiRBC

**Fig. 3:**
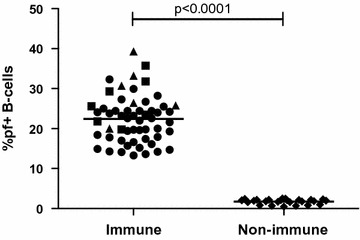
Immune samples from a malaria endemic area (n = 57), and non-immune samples from a non-malaria endemic area (n = 25) where immunophenotyped using carboxyl Qdot-GiRBC, and CD 19. Immune samples had a significantly higher % of falciparum+ B-cells. *Circles* show immune samples but with a negative Rapid Diagnostic Test collected during high transmission season, *triangles* show immune samples with an ongoing malaria infection collected during high transmission season, *squares* show immune samples with an ongoing malaria infection collected during low transmission season and *diamonds* show non-immune samples

Immunophenotyping of *P. falciparum* specific B-cells using carboxyl Qdot-GiRBC and CD 19 PE CF594 fluorochrome-conjugated mouse antihuman mAb was conducted on a total of 57 samples from a malaria endemic area and 25 samples from a malaria non-endemic area. The % of CD19+ cells that were falciparum+ was higher in samples from the malaria endemic area (range 13.3–39.3 %, mean 22.3 %) compared to samples from the malaria non-endemic area (range 0.5–2.5 %, mean 1.7 %), (Fig. 3).

### Reproducibility

**Fig. 4:**
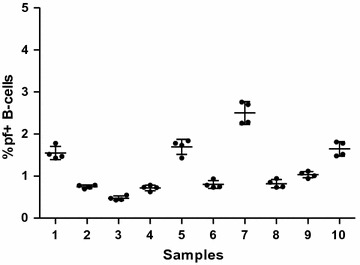
Samples 1–10 from malaria non-endemic area were immunophenotyped four times to test for reproducibility of the assay. Each sample was run 4 times, *lines* indicate mean ± standard deviation

To find out how reproducible the method is, four separate immunophenotyping experiments were conducted (using carboxyl Qdot-GiRBC, and CD 19 PE CF594 fluorochrome-conjugated mouse antihuman mAb) on ten different samples from non-endemic areas using the same conditions. The mean and standard deviation per sample repeat indicated that the method is very reproducible (Fig. 4).

**Fig. 5:**
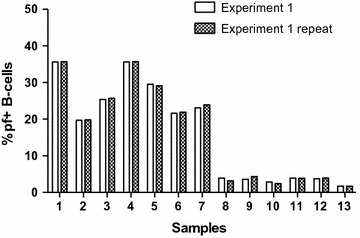
Samples 1–13 were used twice each in repeat experiments to further test for reproducibility of the assay. Samples 1–7 had on going malaria infection, 8–12 had inflammatory conditions other than malaria and 13 was from a non-endemic area for comparison. 1–12 were collected during low transmission season

To further show how reproducible the method is, two separate immunophenotyping experiments were conducted on thirteen different samples, of which seven had a malaria ongoing infection, five were *P. falciparum* negative but with other inflammatory conditions, and one sample was from a non-endemic area as a control (Fig. 5). The samples from the endemic area were all collected during low transmission season. These samples were ran twice only because they had fewer cells, compared to the above non-immune samples. The mean and standard deviation for the two experiments on malaria positive samples was 26.7 (5.8), and 26.8 (5.6), *p* value = 0.43 indicating that the difference is not statistically significant. The mean and standard deviation for the two experiments on inflammatory samples but *P. falciparum* negative was 3.6 (0.4), and 3.5 (0.8), *p* value = 0.81 indicating that the difference is not statistically significant and reproducibility is good, whether high or low values of falciparum+ cells are measured.

### Comparison of falciparum+ B-cells and parasitaemia

**Fig. 6:**
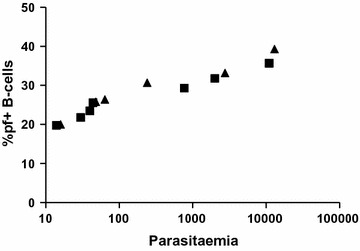
Parasitaemia compared to % falciparum+ B-cells, for samples from immune individuals who had *P. falciparum* parasites in the blood. *Triangles* show samples collected during high transmission season, *squares* show samples collected during low transmission season

All the 57 samples from the malaria endemic area were screened for malaria using the malaria rapid diagnostic test (RDT). Microscopy was done on the 13 samples that where RDT positive to determine the parasitaemia. The plot of % of falciparum+ B-cells and corresponding parasitaemia (Fig. 6) indicated that the higher the parasitaemia, the higher the % of falciparum+ B-cells. There was no obvious difference in results, whether the samples were collected during high or low transmission season (Fig. 6). Within the immune samples (presented in Fig. 3), the mean and standard deviation for the parasite positive (n = 13) and parasite negative (n = 44) samples was 27.9 (6.1), and 20.6 (4.9), *p* value <0.0001.

## Discussion

Quantum dot nano crystals and bioconjugates are ideal for use in flow cytometry, and in this study a novel technique for separating *P. falciparum* specific B-cells from other B-cells in humans has been developed. Different Qdots, and different concentration combinations were tested and found carboxylated Qdots to be the best ones in combination with GiRBC. The removal of haemoglobin from the iRBC to form GiRBC was a major improvement in removing unspecific binding. It was not possible to completely remove unspecific binding, but the differences between immune and non-immune individuals were so large that an estimation of the percentage of *P. falciparum* specific B-cells in peripheral blood can be made. In the individuals with ongoing infection with *P. falciparum*, the levels of B-cells were even higher, indicating that the measure is really a specific response. It is also interesting to note that the higher the levels of parasites in the blood, the higher the levels of falciparum+ B-cells. However, the number of samples with parasites in the blood was quite small, and it needs further studies before any firm conclusions about this can be made. All samples were from adults, and since the “immune” individuals live in Uganda where malaria occurs all year around, they have probably had malaria many times before and they can boost a specific response relatively quickly. It is interesting to note that the samples collected from patients with other inflammatory conditions than malaria (5 individuals) in the low transmission season all showed lower values compared to many of the samples collected earlier in the study during high transmission season. Perhaps other B-cells than falciparum+ B-cells are boosted, making the relative numbers of falciparum+ B-cells lower. For those that had an ongoing infection with *P. falciparum* malaria, the levels of falciparum+ B-cells were in the same range whether collected during the high-or low transmission season (Fig. 6), indicating that an acute infection really boosts falciparum+ B-cells. In future studies, investigations on newborns and small children can be considered to further try and understand the development of *P. falciparum* specific B-cells early in life.

It has for long been a matter of debate, to which extent long-term immunity against different antigens can be boosted when the individual is exposed to other immunostimulatory factors. For malaria, it is quite clear that repeated exposure is necessary to reach a high level of immunity, but it is not yet understood in detail why it takes so long to reach this immunity. Before it has been difficult to study malaria specific B-cells, but if the presently described method is used, it should be easier in future to evaluate specific responses, both in naturally acquired immunity and in vaccine studies. The study capitalized on schizont extract in order to measure all kinds of responses, both against merozoites and iRBC surface molecules. The advantage of this is that high enough levels of B-cells can be found. If B-cells directed only against one specific (recombinant) antigen is looked for, the number of B-cells will probably be too low to be found. However, a combination of antigens might be a way forward in vaccine studies, where specific responses are investigated.

In earlier studies, Elispot assays have been used to try and estimate numbers of *P. falciparum*-specific B-cells. In Elispot, the cells have to survive the experimental environment with activation of the B-cells and transformation into antibody producing cells, and it is difficult to know whether all cells actually persist during this treatment. Several earlier studies have shown conflicting results concerning the correlation between antigen specific IgG levels and memory B-cell frequencies, when Elispot has been used [30, 39, 40]. This indicates that there might be difficulties in the methods that have been used before. It has also been shown that presence of malaria specific B-cells measured by Elispot does not predict protection from a challenge infection [31]. With this new method, no activation of the cells is needed, and because it is based on flow cytometry staining, antigen-specific memory B-cells can directly be detected by virtue of their affinity/avidity for cognate antigen which makes it advantageous.

Despite flow cytometry having high-throughput capabilities, it can be hampered by non specific binding of some antigens especially repetitive or complex multi-protein antigens [41]. Future studies will look into sorting of the positive cells and expression of their antibodies to determine the extent to which they are *P. falciparum*-specific.

## Conclusion

This study has shown that there is a major difference in the percentage of *P. falciparum*-specific B-cells in immune compared to non-immune individuals, with the highest levels in those that have parasites in the blood. Using a new method that opens up new possibilities for evaluating *P. falciparum*-specific B-cells, knowledge about how they are sustained in vivo should be facilitated. This method can hopefully be used in both vaccine studies that are based on infected RBCs or individual antigens that can be conjugated to the carboxyl Qdots, and also for studies of development of naturally acquired immunity.

## Abbreviations

Qdots: quantum dots
PBMC: peripheral blood mononuclear cells
iRBCs: *Plasmodium falciparum* infected red blood cells
GiRBCs: ghost *Plasmodium falciparum* infected red blood cells
falciparum+ B-cells: percentage of CD19+ cells that are positive for *P. falciparum*
falciparum− B-cells: percentage of CD19+ cells that are negative for *P. falciparum*
GiRBC-Qdot: ghost *Plasmodium falciparum* infected red blood cells carboxyl quantum dot conjugate
